# Audio-visual stimulation for visual compensatory functions in stroke survivors with visual field defect: a systematic review

**DOI:** 10.1007/s10072-022-05926-y

**Published:** 2022-02-11

**Authors:** Kholoud Alwashmi, Georg Meyer, Fiona J. Rowe

**Affiliations:** 1 Department of Psychological Sciences, Eleanor Rathbone Building, University of Liverpool, Liverpool, L69 3BX UK; 2 Institute of Population Health, University of Liverpool, Liverpool, L69 3BX UK

**Keywords:** Hemianopia, Audio-visual training, Compensatory training, Multisensory integration, Neuroimaging

## Abstract

**Background:**

Hemianopia is a complete or partial blindness in the visual fields of both eyes, commonly caused by cerebral infarction. It has been hypothesized that systematic audio-visual (AV) stimulation of the blind hemifield can improve accuracy and search times, probably due to the stimulation of bimodal representations in the superior colliculus (SC), an important multisensory structure involved in both the initiation and execution of saccades.

**Methods:**

A narrative synthesis of the findings is presented to highlight how AV rehabilitation impacts on patients with hemianopia including visual oculomotor function, functional ability in activities of daily living, hemianopic dyslexia, visual scanning and searching tasks, maintaining of functional ability post training and the effect on brain multisensory integration by using neuroimaging.

**Results:**

Sixteen studies were included (fourteen articles (188 participants) and two literature reviews). Results were grouped into AV training of hemianopia in adults and in children and then further grouped according to the AV task type: tasks measuring the training effects by comparing visual stimulation training to audio-visual training, localization abilities in homonymous hemianopia (HH) and AV integration in patients with HH.

**Conclusion:**

Systematic AV training may improve the processing of visual information by recruiting subcortical pathways, and because most of the patients with visual cortex damage have an intact SC, it might be useful to use the bimodal AV training to activate retinotectal functions. Nevertheless, the underlying mechanisms supporting the reported positive effects are not currently understood. Systematic functional and/or structural imaging studies may help in understanding the underlying mechanism and inform the design of optimal training paradigms.

**Supplementary Information:**

The online version contains supplementary material available at 10.1007/s10072-022-05926-y.

## Introduction and background

Homonymous hemianopia (HH) is a visual field defect that is defined by complete or partial blindness in the visual fields to the right or left side of both eyes, commonly caused by cerebral infarction [1]. Visual field defects are estimated to affect 20 to 57% of people who have had a stroke [2] with a recent UK reported incidence of 30% [3]. Approximately 8 to 10% of stroke patients with visual field defects have permanent HH [4].

Functional ability in activities of daily living like mobility, reading, driving and general quality of life can be affected by visual field defects following stroke. Additionally, visual field loss may influence the patients’ ability to participate in rehabilitation and has been linked to depression, anxiety and social isolation [2, 5]. There are many interventions for visual rehabilitation: restoring the visual field (restitution therapy), changing behaviour to compensate for lost visual function (compensation training) and substituting for the visual field defect by using a device or extraneous modification (substitution therapy) [6].

Visual scanning training is one of the compensation approaches for hemianopia rehabilitation that has been shown to lead to significant improvements in vision‐related quality of life judgements by affected patients [7–9]. Moreover, previous research shows that rehabilitation using visual scanning training leads to neural changes in addition to compensatory behavioural improvement. A study undertaken by Nelles et al. [10] investigated eye movement training–induced plasticity with functional magnetic resonance imaging (fMRI) in patients with HH and showed increased neural activity in the contralesional extrastriate cortex, suggesting that training of exploratory eye movements induces changes in the cortical representation of hemifields.

It has been hypothesized that audio-visual (AV) stimuli can further enhance saccade precision into the missing hemifield [11]. Furthermore, systematic AV stimulation of the blind hemifield has been shown to improve accuracy and search times in visual exploration, probably due to the stimulation of the superior colliculus (SC), an important multisensory structure involved in both the initiation and execution of saccades [12]. SC areas receive converging projections from different senses and consist mainly of multisensory neurons. As a result, the unimodal processing of visual information in the blind hemifield seems to be affected by the interaction between different sensory inputs occurring within the SC [13]. The process of synthesizing the information provided by our different senses is known as “multisensory integration”, which can improve perceptual and behavioural performance more than the sum of their individual action, leading to a profound impact on our daily lives [14].

Tinelli et al. [15] found that audio cues, spatially and temporally coincident with a visual stimulus, improve visual perception in the blind hemifield of patients with HH. This observation can be explained by populations of neurones in the SC that response ‘super additively’ to bimodal signals that are temporally and spatially coincident. When Wallace et al. [16] examined neuron responses to multisensory cues in the SC of the rhesus monkey, it showed that each of unimodal- and multisensory-responsive neurons was clustered by modality, these modalities were represented in map-like fashion and the different representations were in alignment with one another. Each multisensory SC neuron has multiple receptive fields, and each responds to a different type of stimulation. It has been claimed that the combination of stimuli may result in response enhancement, response reduction or no interaction, depending on the location of the stimuli relative to one another and to their receptive fields, respectively [16]. Consequently, maximal response interactions were seen when visual and auditory inputs originated at the same source position, and thus are likely to be caused by the same event. Stimuli that were spatially separated, in contrast, would fall outside the excitatory borders of its receptive field, so that either no interaction is produced or one stimulus diminishes the effectiveness of the other [16].

The expectation of an enhancement of visual processing is also based on an important and adaptive property of multisensory integration [13]. Repeated experience with the same AV stimulus can increase the neuron’s sensitivity to auditory and visual individual inputs [14], leading to the conclusion that visual and spatial compensatory functions can be reinforced by audio-visual training (AVT) in adult patients with chronic visual field defects following a stroke.

The factors that influence how a person adapts to visual field loss, the interventions that are available to aid the adaptation process as well as the effects of interventions on people with visual field defects after stroke are covered well in previous reviews [2, 6, 17]. AVT-induced plasticity in patients with hemianopia, however, is less well described [10]. Consequently, further research is required to identify the optimal training paradigm and the neural mechanisms underlying the effect of bimodal stimulation (AVT) [18]. This review focuses on the effectiveness of interventions that use AV multisensory training as rehabilitation for stroke survivors with visual field defects and its impact on the quality of their daily life and the underlying change in brain function and structure.

## Methodology

The review is observed and reported according to the Preferred Reporting Items for Systematic Reviews and Meta-Analyses (PRISMA) guidelines [19, 20] (Table 3 in the Appendix). We used the Preferred Reporting Items for Systematic review and Meta-Analysis Protocols (PRISMA-P) 2020 checklist with recommended items to address this systematic review protocol [19, 20] (Table 4 in the Appendix).

### Eligibility criteria

Searching in the databases covered randomized trials, controlled trials, prospective and retrospective cohort studies, observational studies and case–control studies in adults after stroke, where the intervention is focusing on multisensory integration, especially AV training, to improve the visual and spatial compensatory functions or improving the ability of the participant to cope with the visual field loss. Case reports and letters were excluded. Articles that discussed other visual impairments alongside HH but discussed visual field loss separately were included. As most of the articles were in English, we searched without defining a specific language and there were no translations needed. It has been reported that AVT can induce activation of visual responsiveness of the oculomotor system in children and adolescents with acquired lesions as effective as in adults [21]. Therefore, we included studies of adult and child participants reporting on visual field loss. We included outcome measurements of a comparison between unimodal and multimodal stimulations in compensatory recovery, functional performance in daily living activities, visual impairment measures for HH, dyslexia, visual scanning and searching task, effect on brain multisensory integration by using neuroimaging, and AV localization and space perception in hemianopia. Studies reporting on mixed populations must have had 50% or more of subjects diagnosed with hemianopia and data available within this category.

### Information sources and keywords

We conducted a full systematic review of the literature in the Scopus, PsycINFO, ScienceDirect, Web of Science and PubMed databases dating from the start of recorded databases for each information source to June 2020 (Table 1).

**Table 1:** Search terms

Multisensory Multisensory integration Multimodal integration Multimodal stimulation Audio-visual training Audio-visual Bimodal stimuli	Hemianopia Visual field defect Visual impairment Stroke Visual field deficit Rehabilitation Visual perception
OR	OR
AND	

### Selection process

After identifying the titles and abstracts from the search, these were screened through each phase of the review using the predetermined inclusion criteria. The titles and abstracts identified from the search were independently screened by one author (KW), and at least 10% were double checked by a second author (FJR) through each phase of the review. When further information was required for this process, the full paper was obtained and the selection criteria were applied. A subsequent review of the full papers was undertaken to determine which studies should be included. We resolved disagreements at each step by discussion between the two review authors. If a disagreement remained, we sought the opinion of a third reviewer (GM).

### Data extraction for the included studies

A designed form was used for the data extraction process. The data extraction form included all the factors identified by the researchers (KW, FJR) as having potential importance for analysing the effect of the AVT on visual compensatory functions: extent of visual field loss; age and gender; research design, sample size, AV training paradigm and training dose; primary and individual assessments; and primary and individual results.

### Quality assessment

The term “quality” refers to “the degree to which a study employs measures to minimize bias and errors in its design, conduct, and analysis” [22]. The quality of included studies was reviewed using the following checklists:

- An adaptive version of PRISMA was used to assess evidence in review articles [19].
- An adapted version of Consolidated Standards of Reporting Trials (CONSORT) was used for evaluation of the quality of evidence in randomized controlled trials and controlled trials [23].
- Quality assessment using the Strengthening the Reporting of Observational Studies in Epidemiology (STROBE) checklist was performed for the observational studies (available on STROBE statement: available checklists; strobe-statement.org).

### Dealing with missing data

Our strategy was to contact authors of included studies if important data were not available. The reviewed papers contained the relevant data so that no authors had to be contacted.

#### Data analysis

We provided a narrative synthesis of the findings from the included studies, structured around the type of intervention, target population characteristics, type of outcome and intervention content. We found a significant heterogeneity between the included studies; therefore, a meta-analysis could not be undertaken. The results are supported by a summary of findings (table and figure highlights).

1. First is AV stimulation training outcome measures on HH adults/children by a comparison between unimodal and multimodal stimulations in compensatory recovery. The outcome measures involve functional ability in activities of daily living, visual scanning and search tasks, hemianopic dyslexia and the electroencephalographic (EEG) measures on spatial attention for HH. In addition, we included the summary of findings regarding the maintenance of functional ability post training.
2. Second is AV localization and space perception in HH.
3. Third is AV integration in patients with visual field defects.

## Results

**Fig. 1:**
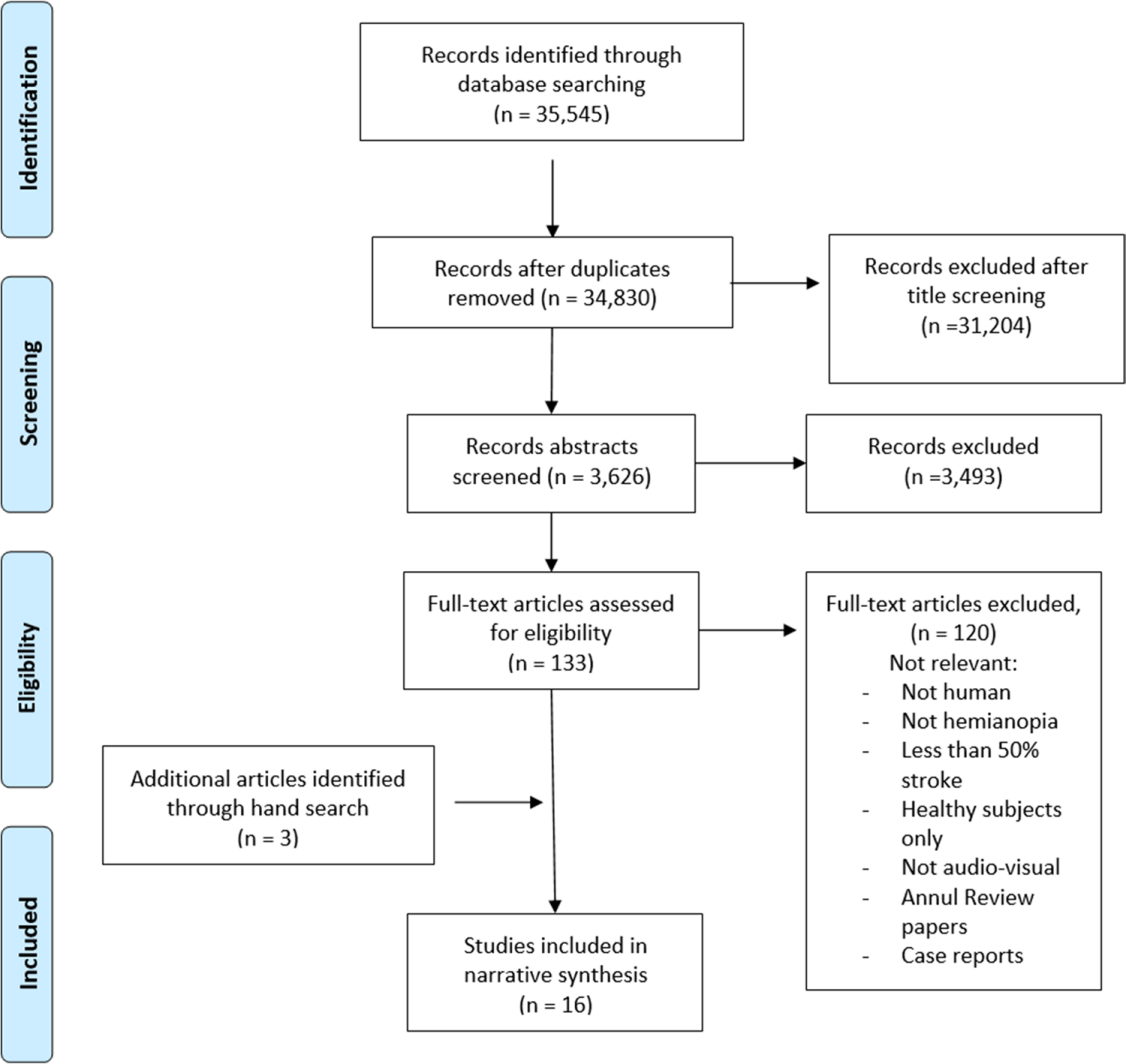
PRISMA flow diagram. Schematic of the literature search and article selection used to identify studies on using AVT as rehabilitation strategy for stroke survivors with HH

The flowchart for this systematic review is illustrated in Fig. 1. Sixteen studies were included (fourteen articles (188 participants) and two literature reviews). The key data extracted from the included studies are presented in Table 2.

**Table 2:** Key data extracted from the studies of audio-visual training for patients with hemianopia

Study ID	Study design	Sample size	Stroke phase	Tasks	Testing method	Training duration	Follow-up	Summary of results
Tinelli et al. (2017) [15]	Uncontrolled longitudinal study	*n* = 3	Chronic, 1 year after the lesion onset	Subjects’ eye movements were recorded under 3 types of stimuli: (1) unimodal visual condition, (2) unimodal acoustic condition and (3) bimodal AV condition. In the bimodal condition, the sound could be spatially congruent or it could appear with a nasal/temporal difference of 16° or 32° (spatially incongruent)	2 visual detection tests, which were performed with the training apparatus (unimodal visual test and bimodal audio-visual test)	The duration of the training program depends on the results reported by the subject on a daily basis and usually lasts from 4 to 6 weeks	Follow-up was performed 6 months after the end of treatment for S1, whilst for S2, it was performed after 12 months, and for S3, after 9 months	The results of the present study confirm the effectiveness of the AVT based on the stimulation of ocular movements and visual exploration functions through compensative strategies. In the eye movement condition, a significant difference was found between baseline and post AVT in all 3 subjects (*p* = 0.008, *p* = 0.004 and *p* = 0.00001, respectively)
Passamonti et al. (2009) [12]	Case–control study	*n* = 24 (12 patients and 12 controls)	Chronic, at least 5 months after the onset of their hemianopia	All the patients underwent control visual training and, subsequently, AVT. In the latter, the 2 stimuli could be presented at either the same spatial position or at positions with a spatial disparity (16° and 32° of disparity); furthermore, the temporal interval between the sound and the light was gradually reduced from 300 to 0 ms over the sessions	Sessions: (S1) initial evaluation, (S2) 2 weeks after S1 through which subjects were performing control VT and (S3) 2 weeks after S2 through which subjects were performing AVT. The assessment consisted of evaluating visual detection ability (performed in fixed-eyes and eye movement conditions), visual exploration (triangle test, number test), reading task and ADL	4 h daily over a period of 2 weeks (2 weeks visual and 2 weeks audio-visual)	3 months later (S4) and, again, 1 year later (S5)	Visual detection and perceptual sensitivity significantly increased in S3 (67%) compared to S2 (47%; *p* < 0.01) The accuracy significantly improved in the triangle test between S2 and S3, S4 and S5 (*p* < 0.003 in all comparisons). Compared to S1, the ADL was significantly reduced at S3 (*p* < 0.001). A significant reduction of the length of scan bath, refixations and search times was obtained in the number test when comparing S2 to S3 (*p* < 0.05)
Bolognini et al. (2005) [26]	Randomized control study	*n* = 8, in 2 groups: G1: baseline 1, baseline 2 and post AVT G2: pre AVT, post AVT and a follow-up	Chronic, more than 4 months after the onset of illness	3 different kinds of sensory stimulation were presented: (i) unimodal visual condition, (ii) unimodal auditory condition (i.e. catch trial) and (iii) cross-modal AV condition, the sound either spatially coincident (SP) or spatially disparate, at 16 and 32 of nasal or temporal disparity from the visual target	All patients underwent assessment of (i) visual detections (unimodal visual test and computerized visual field test), (ii) visual scanning (the E–F test, the triangle test and the number test), (iii) hemianopic dyslexia and (iv) ADL	Daily session lasted 4 h. All patients completed the training in 2 weeks	1 month after the training (4 subjects)	In each group, the difference between the baseline and each AVT session was significant (*p* < 0.0002, in all comparisons). The visual detection assessment: In the unimodal visual test, the interaction condition × session was significant in G1 (*p* < 0.05) and G2 (*p* < 0.005). For the visual exploration and the reading tests, the correct responses and the RTs showed a significant effect of session for both groups. A significant reduction in the ADL scores were found in both groups pre- and post-training (*p* < 0.06)
Keller and Lefin-Rank (2010) [18]	Randomized control study	*n* = 20	Acute, between 3 and 24 weeks primarily after stroke	Visual and acoustic stimuli were presented synchronously in the same spatial positions with a duration of 100 ms. To prevent patients from reacting to false positives, 20% of catch trials with solely acoustic stimulation were implemented in each training session	5 diagnostic tests were administered before and after training. Visual exploration was assessed using the same apparatus, search test, reading test and evaluation of ADL	30 min daily for 3 weeks	N/A	Clear advantage of the AVT in comparison to the VT. Statistically significant differences between both groups for every outcome variable were obtained. In the exploration task, the detection rate of target stimuli improved by about 46% in patients of the AVT group compared to 16% in patients of the VT group. The AVT group nearly doubled the saccades into the blind field compared to 11% only in the VT group, and increased the average amplitude by 12° compared to 4° in the VT group
Lewald et al. (2012) [28]	Cohort study	*n* = 10	Chronic, at least 5 months after the stroke	High-frequency stimulation protocol (10 Hz) for PAS was used with 2 loudspeakers at 76° and 14° to the left and 2 loudspeakers at 14° and 76° to the right. 2 stimulus locations were presented simultaneously. Then, visual stimuli were presented at the lower edge of the chassis of each loudspeaker and RTs were measured	By using the visual detection task: After pre-PAS block 1 was completed, the patient was allowed to rest, and pre-PAS block 2 was started 1 h after the beginning of pre-PAS block 1. The PAS period laid between pre-PAS-2 and post-PAS blocks. After the post-PAS block was completed, the patient was allowed to rest for about 1.5 h. Finally, the recovery block was done	In the visual detection task: The overall duration of each block, including the discarded trials, was about 30 min. PAS were presented over a period of 1 h	The recovery block started 2 h after the end of the PAS period	The percentage of correct visual detections in the post-PAS block was increased by 86.5% with reference to the mean of the pre-PAS and recovery measurements. 1-time passive auditory stimulation on the side of the blind, but not of the intact, hemifield induced an improvement in visual detections. This enhancement in performance was reversible and was reduced to baseline 1.5 h later
Grasso et al. (2016) [27]	Cohort study	*n* = 10	Chronic, more than 3 months after the lesion	Patients were presented with 3 different kinds of sensory: (i) unisensory visual (UV; 100-ms red LED light), (ii) unisensory auditory (UA; 100-ms, 80-dB white noise) and (iii) multisensory AV simultaneously at the same location	Measuring visual detection (unisensory visual test), visual scanning (E–F test, triangle test), reading abilities and ADL. EEG measures were collected at 4 time points: baseline 1 (i.e. before treatment (B1)), control baseline 2 (i.e. 2 weeks after B1 and immediately before treatment (B2)), immediately after treatment (P) and in a follow-up session	4 h daily for 2 weeks (10 days)	Mean time of 8 months after the training	After audio-visual training, improvements in visual search abilities, visual detection, ADL (comparing pre- and post-training *p* = 0.003) and oculomotor parameters (e.g. number of fixations significantly reduced at post AVT *p* = 0.044) were found. For the unisensory visual test, the accuracy scores significantly increased from pre-AVT (37.6%) to post-AVT (66.4%; *p* = 0.0002)
Ten Brink et al. (2015) [29]	Uncontrolled longitudinal study	*n* = 8	Chronic, at least 26 months after lesion onset	2 experiments were performed with 3 testing conditions: (1) unimodal (auditory), (2) bimodal coincident (temporally and spatially) and (3) bimodal disparate (temporally coincident but spatially disparate)	Testing performed by observation throughout the experimental sessions	1st: practice session of 40 trials, 120 unimodal trials, 120 bimodal coincident trials and 360 bimodal disparate trials 2nd: 2 blocks of the experiment consisted of a practice session of 32 trials and an experimental session of 480 trials	N/A	In all 7 hemianopic patients, saccade accuracy was affected only by visual stimuli in the intact (*p* < 0.001), but not in the blind visual field (*p* > 0.05). They concluded that their results show that multisensory integration is infrequent in the blind field of patients with hemianopia. In 1 patient with a more limited quadrantanopia, a facilitation effect of the spatially coincident visual stimulus was observed (*p* = 0.002)
Lewald et al. (2013) [34]	Case–control study	*n* = 20 (10 patients and 10 control)	Chronic, more than 6 months after the onset of illness	In experiment 1, subjects had to bring a visual stimulus into spatial alignment with a target sound. Each trial began with the onset of the sound stimulus at 1 of the 21 positions. In experiment 2, subjects were asked to bring an acoustic stimulus into spatial alignment with a visual target	The assessment has been done through the 2 experiments	Each experiment comprised 168 trials plus repetitions (8 presentations of each stimulus position)	N/A	The bias of visual pointing, with reference to auditory target towards the anopic side, was stronger within the anopic hemispace (mean 6.72°) than intact hemispace (mean 2.96°) (*p* = 0.024). The bias of acoustic pointing was almost equal in anopic (mean 4.39°) and intact hemispaces (mean 3.83°) (*p* = 0.44). The auditory space of patients with pure HH remained largely unaffected by the consistent AV disparity
Dundon et al. (2015) [31]	Cohort study	*n* = 8	Chronic, at least 3 months after the lesion	(i) UV, (ii) UA and (iii) multisensory AV (MAV) condition (UV and UA simultaneously at the same location). Patients detected the presence of a light stimulus presented on the horizontal meridian, by pressing a button. The visual stimuli could appear at 1 of 8 eccentricities (56°, 40°, 24° and 8° bilaterally)	Patients completed both a clinical assessment and an EEG paradigm at 3 time intervals—baseline 1 (i.e. before treatment (B1)), control baseline 2 (i.e. 2 weeks after B1 immediately before treatment (B2, control for practice effects)) and finally after treatment (P). Unisensory visual test, visual scanning (E–F test, triangle test, number test) and ADL were performed	10 days; 4 h of training per day	N/A	A main effect of session on the hemianopic field was revealed (*p* < 0.001); accuracy scores significantly increased from B1 (34%) and B2 (39%) to P (71%, *p* < 0.001). The present results suggest that both visual exploration and attentional allocation towards the blind field might collaborate to help compensate for visual field defects and to improve daily quality of life of patients with HH, and the ADL scores were significantly lower in the post AVT (7) compared with B1 and B2 (11, *p* = 0.002)
Leo et al. (2008) [32]	Uncontrolled longitudinal study	*n* = 12	Chronic, > 2 months after onset	Experiment 1 tested the effect of spatial coincidence between visual and auditory stimuli. Experiment 2 tested whether this effect also depended on temporal coincidence. Both experiments used the same apparatus. 3 testing conditions were used: (1) unimodal auditory condition, (2) unimodal visual catch-trial condition and (3) cross-modal condition	The assessment has been done through the 2 experiments	The total number of trials was 600, and these were equally distributed in 15 experimental blocks (40 trials each) over 2 consecutive days	N/A	In the hemianopic field, a spatially and temporally coincident AV stimulus significantly reduced the localization error established in the unimodal condition (10° vs. 13°, *p* < 0.02, respectively). This multisensory benefit was as great in the blind field as in the intact hemifield. A significant main effect was obtained only for hemifield in the visual bias of sound location, showing that only the intact field did the visual stimulus bias sound localization (visual bias in the intact = 41%, visual bias in the blind = 1%)
Lewald et al. (2009) [35]	Case–control study	*n* = 24 (12 patients and 12 control)	Chronic, at least 5 months from illness onset	2 blocks were conducted subsequently in fixed order. In the first block, subjects adjusted a visual stimulus towards their subjective straight-ahead direction. The second block was conducted in exactly the same way as the first block, but with presentation of acoustic stimuli from loudspeakers instead of visual stimuli. The subjects were instructed to align the sound position with their felt straight-ahead direction	The assessment has been done through the experiment	2 blocks each contains 63 trials	N/A	The results indicate that in hemianopia, the subjective straight ahead is unaffected. The actual straight-ahead position (0°) has a significant leftward bias of visual straight ahead in LHH (*p* = 0.016) as well as in healthy control (*p* = 0.017), but not in RHH (*p* = 0.46), whereas auditory straight ahead was insignificant in all groups. This suggests that visual brain areas, as are damaged in hemianopia, are not directly involved in relating body position to the external space
Frassinetti et al. (2005) [13]	Uncontrolled longitudinal study	*n* = 21 (7 HH, 7 neglect and 7 neglect with HH)	Not specified	In each trial, 3 different combinations of visual and auditory stimuli could be presented: (1) unimodal visual condition, (2) unimodal auditory condition (i.e. catch trials) and (3) cross-modal condition, spatially coincident or disparate. The auditory and the visual stimuli were simultaneously presented	The number of visual detections made in unimodal condition to those made in cross-modal conditions was compared in patients with neglect (N + H), with hemianopia (NH +) and with both neglect and hemianopia (N + H +)	Each session lasted approximately 2 h and was run on 2 consecutive days	N/A	These results showed cross-modal effects in neglect patients without hemianopia and in hemianopic patients without neglect, but not in patients with both. In both nasal and temporal positions, the detection accuracy increased in both HH and neglect patients with coincident AVT (*p* < 0.02 and *p* < 001, respectively) but not in patients with both defects (*p* ≥ 0.88). A negative correlation was found between the number of cortical areas involved by lesion and the magnitude of multisensory enhancement (*r* = 0.78, *p* < 0.004)
Passamonti et al. (2009) [33]	Uncontrolled longitudinal study	*n* = 15 (9 HH and 6 neglect)	Chronic, at least 5 months from illness onset	In experiment 1, the adapting stimuli were spatially disparate and consisted of a sound coming from straight ahead (0) and a discordant visual stimulus presented at 7.5° from the midline in either the normal or the affected field, in 2 separate blocks. In experiment 2, the adapting stimuli were spatially coincident and consisted of an AV stimulus pair presented at 20° from the midline in either the normal or the affected field, in 2 separate blocks	Unimodal visual detection task: A visual target was presented for 100 ms in each of 4 spatial positions (7.5° and 20° left and right of the fixation point). Patients were asked to press 1 of 2 response buttons to indicate the presence or absence of the visual target	1 day, brief exposure to AV stimuli, each exposure phase lasted 4 min	N/A	After exposure to spatially disparate stimuli in the normal field, all patients exhibited the usual shifts towards the visual attractor, at each sound location. In contrast, when the same kind of adaptation was given in the affected field, a consistent shift was still evident in neglect patients (*p* = 0.53) but not in patients with hemianopia (*p* < 0.0001). After adaptation to spatially coincident stimuli, and regardless of the adaptation hemifield, all patients exhibited a significant improvement in auditory localization
Tinelli et al. (2015) [21]	Uncontrolled longitudinal study	*n* = 3	Chronic, 1 year or more from lesion onset	3 sensory stimulations were presented: (i) unimodal visual condition, (ii) unimodal auditory condition and (iii) cross-modal visual-auditory condition (spatially coincident or spatially disparate). Treatment started with 500 ms between the 2 stimuli, i.e. the auditory stimulus preceded the visual target by 500 ms, and it was reduced in steps of 100 ms	Assessment of (i) the correct number of visual detections (unimodal visual test, using the same apparatus under 2 conditions: eye movement and fixed-eyes), (ii) visual search abilities and (iii) reading speed	Daily session lasted about an hour and half, and the duration of training lasted from 3 to 4 weeks	1 month after the training (3 subjects) and 1 subject after 12 months	The authors found a marked improvement in detections and RTs only when subjects use explorative eye movements (*p* = 0.016), but not with fixed eyes. In the visual search tests, a significant effect was found between baseline and post training, mean value (*p* = 0.0001) and 1 month later (*p* = 0.0017)

*HH* homonymous hemianopia, *AVT* audio-visual training, *VT* visual training, *ADL* activities of daily living, *PAS* passive auditory stimulation, *EEG* electroencephalographic, *RT* response time, *SC* superior colliculus

### Quality assessment

The risk of bias was assessed for each of the included articles (Online Resources 1, 2 and 3) (see additional Supplementary Files 1, 2 and 3). Overall, no article scored 100% for quality assessment in this section. The articles included 12 observational studies, two randomized controlled studies and two reviews. Twelve of the 16 articles scored between 76 and 87% on qualitative assessment and were deemed to have good quality. Four studies scored between 52 and 73% on the relevant quality checklists. All articles were included in this review.

## Adult populations

### AVT outcome measures on patients with hemianopia

#### Visual training vs. audio-visual training

Two reviews discussed visual rehabilitation using multisensory stimulation to compensate for the visual loss after stroke [24, 25]. Seven original research articles recruited patients with HH and trained them on AVT to study the effects of multisensory training on oculomotor scanning behaviour in comparison to the visual training (VT). A total of 71 patients (and 36 controls) were recruited for the AVT and tested on the same apparatus before and after the training period, either on unimodal visual detection task or on both unimodal and multimodal visual detection tasks. These included the following study types: two randomized controlled studies (*n* = 32) [18, 26], two cohort studies (*n* = 40) [27, 28], one case–control study (*n* = 24) [12] and two uncontrolled longitudinal studies (*n* = 11) [15, 29]. The training duration varied between 2 weeks and 2 months, except in one study where one practice and one experimental session were undertaken, and the evaluation was performed during the experiment [29].

During the training, visual and auditory stimuli were presented in four different ways:

1. Spatially and temporally congruent only, by presenting the same duration of acoustic and visual stimuli (100 ms) at the same time and in the same spatial position, in two studies [18, 27]
2. Spatially congruent and disparate with temporally congruent only, in which the acoustic and visual stimuli were presented either at the same time and location or in the same time but different locations (16° and 32° of disparity in either side), in two studies [15, 29]
3. Spatially and temporally congruent and disparate, in which the spatial disparity between the acoustic and visual stimuli was systematically varied (0°, 16° and 32° of disparity) and the temporal interval between the acoustic stimulus and the visual target was gradually reduced from 500 to 300 to 0 ms, in two studies [12, 26]
4. Passive auditory stimulation which depends on the hypothesis that sensory input from an intact modality (auditory) may improve processing of information by spared structures of a damaged sensory system (visual) through synchronous neural activity by repetitive sensory stimulation without requiring any active task from the patient; this protocol is referred to as coactivation or unattended activation-based learning, in one study [28]

All studies reported an improvement in ocular exploration after AVT, which allowed patients to efficiently compensate for the loss of vision with a clear advantage of the AVT in comparison to the visual-only exploration training. It has been found that a sound, spatially and temporally coincident to a visual stimulus, can improve visual perception in the blind hemifield of patients with HH [24, 26].

Imaging studies in humans have confirmed the involvement of the SC and posterior cortical areas, including the temporo-parietal and posterior parietal cortices, in mediating AV multisensory integration [24]. The studies suggested that, because most of the patients with damage to the visual cortex have an intact SC, it might be possible to train the use of retinotectal functions by AV stimulation in both chronic and acute phases after the stroke [12, 18, 24]. Nevertheless, Ten Brink et al. [29] indicated that saccade accuracy was affected only by visual stimuli in the intact, but not in the blind visual field for all patients with HH participating in their study and, only in one participant with a more limited quadrantanopia, was an enhancement in the oculomotor eye movement after the spatially coincident visual stimulus was observed. They concluded that multisensory integration is infrequent in the blind field of patients with hemianopia.

Conversely, in the study of Jay and Sparks [30], trained monkeys made saccadic eye movements to auditory or visual targets whilst monitoring the activity of visual-motor (VM) cells and saccade-related burst (SRB) cells. The authors stated that “the SC is a site where sensory signals (either auditory or visual signals), originally encoded in different coordinates, converge and are translated into a common motor command: a command to correct for saccadic motor error” [30]. This was based on the largely accepted basic hypothesis that sensory input from an intact modality (audition) can enhance processing of information by spared structures of a damaged one (vision). Lewald et al. [28] assumed that AV bimodal neurons not only react to stimulus combinations and integrate information from different sensory inputs, but also can respond to unimodal stimuli and provide a substrate for signalling in two separate modalities. It has been shown that one-time passive auditory stimulation on the side of the blind, but not of the intact, hemifield of patients with hemianopia induced an improvement in visual detections by almost 100% within 30 min after stimulation [28]. The authors assumed that an activation of the surviving parts of the primary pathway and/or the colliculo-pulvinar-extrastriate pathway in HH may lead to an improvement of the related residual visual abilities in the blind field, either by more effective sensory processing of unimodal visual information within the residual pathway or by an increase of spatial attentional functions.

Head fixation and eye movement were monitored in all the studies. Five studies used an optic eye tracker (Eye-Track ASL-6000) and/or infrared video camera where the position of the subject’s eye in the visual scene was monitored on-line by the experimenter [12, 15, 18, 27, 29]. Two studies analysed the eye movement using electro-oculography (EOG) [18, 28]. Fixation was monitored visually by the experimenter standing behind the apparatus in one study [26]. Improvement in oculomotor exploration characterized by fewer fixations and refixations, quicker and larger saccades, reduction in scan path length and the mean exploration time was reported in five studies [12, 15, 18, 27, 28].

All studies demonstrated that the improvement found can be ascribed to compensatory behaviour as there was no significant difference observed in perceptual sensitivity when patients were not allowed to move the eyes (fixed-eyes condition), emphasizing that the treatment did not improve the scotoma in the visual field. This means that AVT is not a restorative treatment in nature.

#### Activities of daily living

Three articles evaluated the activities of daily living (ADL) for HH (total *n* = 30 [8, 10, 12]) in the chronic stage, more than 3 months after stroke [12, 26, 27]. A self-evaluation questionnaire containing 10 items, on a 5-point Likert scale was used including (1) seeing obstacles, (2) bumping into objects/obstacles, (3) losing the way, (4) finding objects on the table, (5) finding objects in the room, (6) finding objects in the supermarket, (7) walking in a crowd, (8) reading, (9) to go upstairs/downstairs on the staircase and (10) crossing the streets. Evaluation of ADL for stroke survivors with HH in the subacute stage, between 3 and 24 weeks after stroke, has been reported in one study (*n* = 20) [18]. The questionnaire comprised only items that can be observed in an inpatient rehabilitation setting including finding objects on the table, avoiding bumping into objects/persons, eye contact, seeing obstacles and reading.

AVT promoted a significant reduction in subjective perceived disability according to the analysis of ADL for patients in both chronic and acute stages after brain damage, confirming a transfer of training effects to ecological environments. It has been indicated that whilst there was a significant improvement in the ADL results after the AVT, no difference was observed after a control VT, consisting of systematic visual stimulation of the visual field on the same apparatus as the AVT. The control VT was performed for 2 weeks before starting the AVT and for a similar amount of training time (4 h/day) [12].

#### Visual scanning/searching

Visual scanning or visual search tests consisted mainly of three subtests: (1) the ‘E–F test’, where patients search for the letter F embedded among distractors, the letter E; (2) the ‘triangle test’ where patients reported the number of triangles embedded within square distractors with the same size and colour; and (3) the ‘number test’ containing 15 numbers (from 1 to 15) randomly distributed over a black background from which the patient is asked to point to the numbers in an ascending order.

Findings provided by four articles (*n* = 50, 32 males and 18 females) reported a significant improvement in visual search performance both in terms of accuracy and search times. Patients’ visual scanning behaviour became more efficient and faster, by comparing pre- and post-AVT tests, giving evidence that stimulating the SC may induce a more organized pattern of visual exploration due to an implementation of efficient oculomotor strategies [12, 18, 26, 27]. Passamonti et al. [12] observed significantly fewer fixations, saccadic duration was reduced and mean saccadic amplitude was significantly increased in all comparisons before and after AVT. Additionally, in a study conducted by Keller and Lefin-Rank [18], the detection rate of target stimuli improved by about 46% in patients of the AVT group, whereas in patients of the VT group, it only improved by 16%. This may suggest that the amelioration in visual perception induced by training is mostly mediated by the oculomotor system where patients can actively scan via eye movement [26], supporting the hypothesis suggested by [30] that auditory and visual signals have been translated into common coordinates at the level of the SC and share a motor circuit involved in the generation of saccadic eye movements.

#### Hemianopic dyslexia/reading

Assessment of hemianopic dyslexia has been undertaken in four articles before and after the AVT (*n* = 50, 32 males and 18 females). In one study, the reading task was for single words only, which were presented in upper-case Italian letters [26]. The other three studies examined the reading time and accuracy by using longer texts (8 to 20 lines) [12, 18, 27]. Comparisons between VT and AVT revealed statistically significant differences in favour of bimodal training. The reading time for patients in the AVT group reduced from 177 s before training to 75 s after training. However, a slight reduction in the reading time was shown for patients in the VT group (from 195 s initially to 175 s post training) [18]. In addition, Grasso et al. [27] revealed a significant effect of the AVT on the reading speed of patients with HH. Generally, according to a review on visual rehabilitation comparing multisensory stimulation and visual scanning, the reading performance improved in all patients after the AVT treatment period, reducing the ocular reading parameters for both progressive and regressive saccades [24]. Lateralization effect on reading impairment was observed in regard to the affected hemifield in patients with HH, by measuring five variables: the number of saccades in the reading direction, the number of regressions (backward saccades), the number of saccades during the return sweep (additional to the one necessary to start a new line), the mean duration of fixation and the amplitude of reading saccades, in only one study [12]. For right HH, the saccadic amplitude increased and the fixation duration reduced during reading, with fewer errors, fewer progressive saccades and fewer regressions, whilst only the number of saccades during return sweep decreased in left HH [12]. Patients with left HH obtained an almost complete normalization of defective ocular responses; however, patients with right HH still showed an impairment of the ocular responses, despite the clear benefit gained [12].

### Electroencephalographic measures on spatial attention for HH

**Fig. 2:**
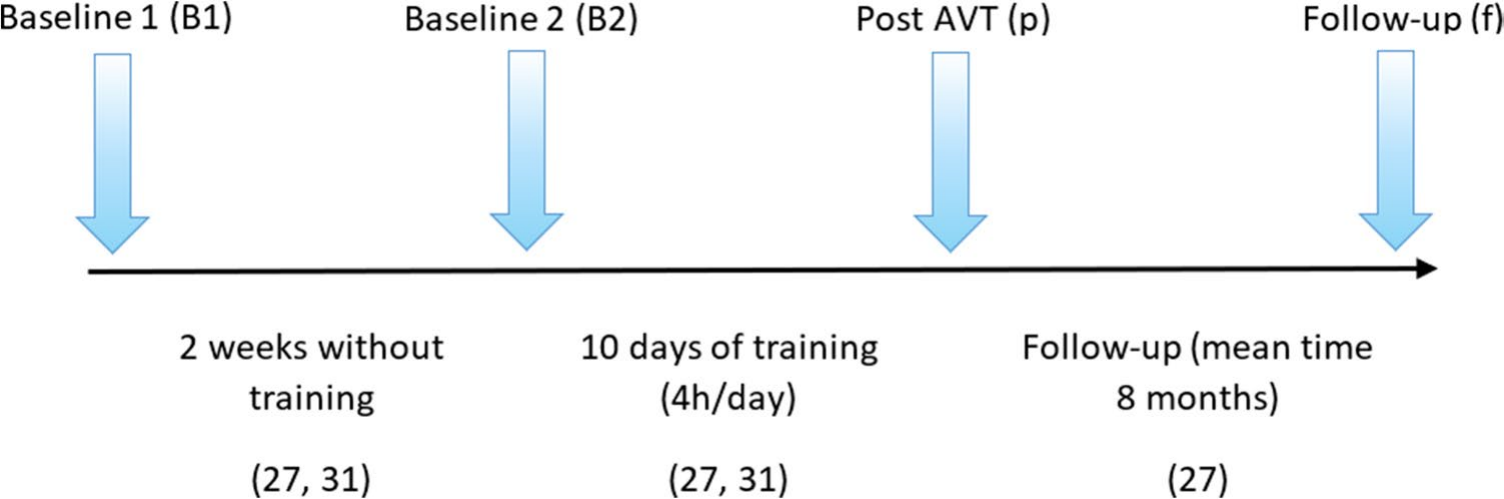
The timeline for the EEG measurements in two studies [27, 31]. Clarification of the sessions where the EEG measurements were obtained over time

A similar study paradigm was used in two studies for the EEG assessment, in which patients performed a simple visual detection task. EEG data were recorded in both studies (*n* = 18, 15 males and 3 females) [27, 31] at three time points: baseline (B1), 2 weeks after B1 and immediately before the AVT (control baseline B2) and after the AVT treatment (p). In addition, Grasso et al. [27] included a follow-up session (f) 8 months after the treatment ceased (Fig. 2).

**Fig. 3:**
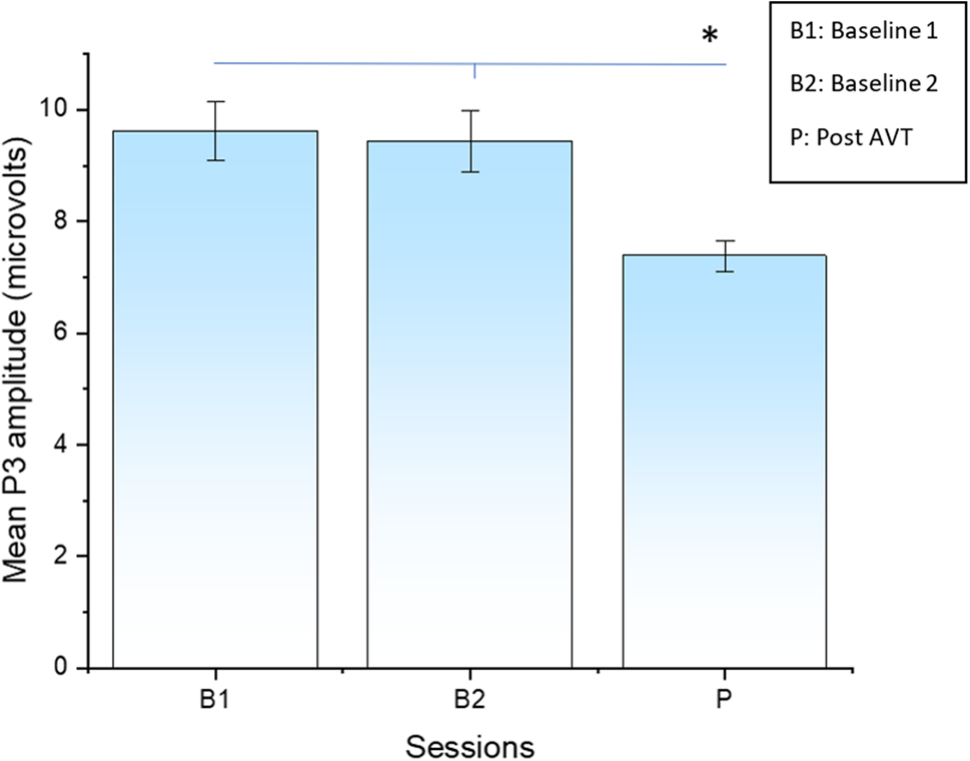
Mean P3 amplitudes. Calculated as a mean value of P3 amplitude results of Grasso et al. [27] and Dundon et al. [31], measured as a function of testing session (B1, B2. P). Asterisks connected with lines indicate significant comparisons (*p* < 0.05)

The data was recorded from 27 electrode sites and the right mastoid. The left mastoid was used as reference, whilst the ground electrode was positioned on the right cheek [27, 31]. P3 components were measured as the mean amplitude in a time window between 200 and 600 ms after the presentation of the stimulus. In the chosen time window, scalp topography at B1 indicated a maximal positive inflection over electrodes CP1, P3 and Pz [27, 31]. Therefore, data from these electrodes were used for statistical analysis. Dundon et al. [31] computed the P3 amplitudes separately for the left and the right HH groups. The average value of electrodes that fell within each group’s zone of maximal P3 amplitude from the individual group topographies was used, i.e. Pz, P3 and CP1 for the right lesion group, and P4, CP2, C4 and CP6 for the left lesion group. A reduction in P3 amplitude in response to stimuli presented in the intact field was reported in both studies, indicating reallocation of spatial attention resources after AVT (Fig. 3). The EEG results obtained by Grasso et al. [27] and Dundon et al. [31] showed that the mean P3 amplitude at session P (7.38 µV) was significantly lower compared to the mean P3 amplitude at B1 (9.62 µV; *p* < 0.05) and at B2 (9.435 µV; *p* < 0.05). No significant difference, however, in P3 amplitude was recorded between B1 and B2 (Fig. 3). In the follow-up session, Grasso et al. [27] reported that the mean P3 amplitude at session F (7.99 µV) was also significantly lower than the mean P3 amplitudes at B1 and B2. A reduction in the intensity of cortical processing in the contralesional hemisphere manifests an improvement in the dynamic visual performance, specifically in the hemianopic field, which indicates attenuation of the allocation of attention towards the intact hemifield. Dundon et al. [31] concluded that multisensory stimulation may significantly reduce the ipsilesional attentional bias in patients with HH.

To test possible different contributions of the left and right hemispheres to the resulted P3 reduction in the study of Dundon et al. [31], only the more lateralized electrodes CP1 and P3 were considered, with group as a between-subjects factor (left lesion patients vs. right lesion patients) and with electrode (CP1, P3), session (B1, B2, P) and position (upper, middle, lower) as within-subjects factors. A significant effect of session (*p* = 0.029) was found with no significant effect of group or any significant interaction between groups and the other factors. These results suggested that no considerable difference of the P3 amplitude reduction was found between left and right HH patients in post AVT training. So, the observed reduction in attention towards the intact hemifield, which might co-occur with a shift of spatial attention towards the blind field, happens similarly in both hemispheres [31].

#### Change over time

Assessment of the intervention effects over a prolonged period of time is of importance to consider treatment effectiveness [25]. Four articles (*n* = 45, 28 males and 17 females) incorporated a follow-up test post AVT training in their design at a period between 1 month and 1 year [12, 15, 26, 27]. There was a transfer of AVT treatment gains to functional measures assessing visual field exploration and to daily life activities which were found to be stable at follow-up control sessions in all the studies, indicating a long-term persistence of treatment effects on the oculomotor system. These long-lasting effects according to Grasso et al. [27] are most probably subserved by the activation of the spared retino-colliculo-dorsal pathway, which boosts orienting responses towards the blind field, increasing the ability to both compensate for the visual field loss and concurrently attenuate visual attention towards the intact field.

Nevertheless, in the study conducted by Lewald et al. [28], ten patients with pure HH received 1 h of passive auditory stimulation by application of repetitive trains of sound pulses. Immediately before and after the auditory stimulation as well as after a recovery period of 2 h, they completed a simple visual task (see visual training vs. audio-visual training section for more details). Whilst the visual detection improved immediately post auditory activation, after the recovery period, the enhancement in performance had returned to baseline, showing that the improvement in performance is not long-lasting when passive auditory stimuli were used.

### Audio-visual localization and space perception in hemianopia

Four observational studies (*n* = 43, 29 males and 14 females) investigated AV localization [32, 33] and the geometry of the visual space in HH using multisensory stimuli [34, 35]. The former studies predicted that visual stimuli in the intact visual field would bias the auditory localization, so that sounds would be mislocated towards their visual source. On the contrary, they expected that in the blind field, where the occipital cortex damage had disrupted its underlying neural circuit, this effect of bias would not occur [32]. Both studies examined the cross-modal condition in which the auditory stimuli were presented with visual stimuli in either spatially coincident or spatially disparate. The results in the intact visual field were in line with the phenomenon known as the ventriloquism effect. In this effect, a presentation of auditory and visual stimuli that are temporally coincident and spatially disparate might lead to mislocation of sounds towards their visual source [36]. In the hemianopic field, however, no visual bias occurred when the two stimuli were spatially separated, which supports the key role of visual cortex for such an effect, so that, when the visual cortex has been damaged, no visual bias was observed [32].

This is because the enhancement of auditory localization is expected via SC neurons, depending on the multisensory activation. It has been shown that visual stimuli affected auditory localization only when stimuli were spatially and temporally coincident, meaning that covert visual processes remain active in hemianopia [32]. The authors explained the difference between the enhancement in multisensory stimulation and the visual bias as these two results are dependent on different neural pathways. The multisensory stimulation is dependent on circuits that involve the SC which facilitate orientation and localization of cues from multiple senses, and the visual bias is dependent on geniculo-striate circuits that provide analysis of the visual scene [32]. A similar result was obtained by Passamonti et al. [33], by comparing patients with HH and patients with neglect (*n* = 9 and *n* = 6, respectively). A consistent shift in sound localization towards the visual attractor was still evident in patients with neglect but not in patients with HH, supporting the role of the geniculo-striate circuits, which is damaged in HH, in such an effect.

The latter studies investigated the concept of how unilateral brain damage in HH can affect the perception of body orientation in space, leading to an attentional bias towards the contralesional field. Lewald et al. [35] indicated that auditory spatial orientation in HH, without spatial neglect, was almost normal compared with healthy subjects. Thus, it was suggested that in multimodal space, visual brain areas, as are damaged in HH, are not directly involved in relating body position to the external space [35]. Additionally, subjects were asked to match the location of a single visual target with an auditory marker or vice versa to estimate the potential distortions in the representation of visual space accompanied by HH [34]. It has been highlighted that patients with HH may exhibit distortion in both the visual and auditory spaces. However, in the bimodal approach, they would cancel each other out, and as a consequence, the cross-modal abilities might be preserved [34].

### Audio-visual integration in patients with visual field defects

The anatomical correlation of audio-visual integration was investigated by a comparison between patients with hemianopia and patients with spatial neglect in two studies (*n* = 36, 22 males and 14 females) [13, 33]. Both studies showed that after adaptation to spatially coincident AV stimuli, both HH patients and neglect patients exhibited significant reduction in auditory and visual localization errors. A possible explanation for these effects is the function of multisensory neurons in the SC, which can be activated when the stimuli from different sensory modalities at close spatial proximity interact [13]. Thus, the results indicated that damaged brain areas (striate and parieto-temporal areas) in HH and neglect patients were not contributory in this specific form of perceptual learning [33]. In other words, visual information is capable of calibrating auditory space, even without the involvement of those brain areas, as long as visual information and auditory information are spatially coincident. Passamonti et al. [33] found that adaptation to spatially disparate stimuli invokes the geniculo-striate circuit to correct and reduce the discrepancy registration. However, adaptation to spatially aligned stimuli invokes the collicular-extrastriate circuit to reduce the localization errors. Therefore, the multisensory enhancement should be observed in both neglect and HH patients as the collicular-extrastriate circuit is spared in both patients [33].

By contrast, in patients suffering from hemianopia and neglect, multisensory integration did not occur [13]. It has been reported that integrative multisensory effect depends on the extension and/or the localization of the lesion. Lesions causing neglect are mainly confined to the frontotemporal and parietal lobes (visuospatial attentional system) whilst lesions causing HH are mainly confined to the occipital lobe (the primary sensory visual system), and for patients with both neglect and HH, the lesion could involve both areas [13]. A possible explanation provided was that the influence of these cortical areas modulates the ability of SC to synthesize cross-modal inputs, preventing the cross-modal integration in patients with both deficits.

## Childhood population

The possibility of inducing long-lasting amelioration after AVT in children with chronic HH due to acquired brain lesions was investigated by only one study [21]. The study included three children (one male and two females aged between 9 and 17 years). The training duration was one and a half hour daily for 4 weeks. Outcome measures consisted of correct number of visual detections, visual search ability and reading speed. The visual search test consisted of six different subsets: the apple, frog, smile, E–F, triangle and number tests [21]. Each subject was tested before and after the training period and after a follow-up period of 1 month, and in one case, further follow-up was obtained after 12 months. The authors found a marked improvement in detections and response times only when subjects used explorative eye movements, but not with fixed eyes on a central point [21]. This suggests that the enhancement in visual perception induced by training is mediated by the oculomotor system, reinforcing orientation towards the blind hemifield. For all the tests, the main factor session was significant when response times were considered [21].

Improvement in reading speed after training was observed for the single word reading performance for all subjects. The results of this study confirmed that AVT can also induce activation of visual responsiveness of the oculomotor system in children and adolescents with visual field deficits as the visual search behaviour became more efficient and faster after treatment. Tinelli et al. [21] argued that this manifests the important role of the multisensory integration especially the SC in this type of ocular compensation and in the plasticity of the visual system in the presence of ‘blindsight i.e. residual visual capacity but without acknowledged perceptual awareness after lesions of the striate cortex’ even when the occipital cortex is completely damaged. Long-lasting effect of the treatment was reported in both 1-month and 12-month follow-up tests [21].

## Discussion

This review assessed the effectiveness of AV stimulation as a rehabilitation option for stroke survivors with HH in adulthood and childhood. The included studies suggested that unilateral damage to the visual cortex of the occipital lobe may retain behavioural responses to visual stimuli in the lost visual field, with the existence of blindsight effect, especially when combined with acoustic stimuli. Neurophysiological and neuropsychological studies have discussed that the spared striate cortex, the extra-striate visual cortex and neural pathways involving subcortical nuclei such as the SC could mediate the blindsight effect [37, 38]. Although blindsight could be considered as an implicit process occurring without an explicit knowledge, Frassinetti et al. [13] argued that even when patients with HH were aware of the presentation of visual stimuli in the blind hemifield, there was an improvement in the processing of the visual detection in the multimodal stimulation, suggesting another interpretation of this finding. They have suggested that the responses probably are mediated by cortical areas (poly-sensory and/or sensory-specific cortices) that might be multimodal areas, and are involved in the cross-modal integration.

Several studies provide evidence showing that multisensory integration through the SC is expected to have enhanced the responsiveness of the oculomotor system by reinforcing the orientation towards the affected hemifield. The SC is a midbrain structure that receives visual, auditory and somatosensory inputs, and is involved in detecting, locating and orienting to external events [14]. Thus, the sensory information from an unimpaired modality (auditory) might improve the processing of information from an impaired sensory system (visual) [25]. The functional properties of multisensory neurons have been most extensively studied, since the last few decades, in the cats’ and monkeys’ SC [14, 37], yet its organization follows a general mammalian scheme. Stein and Rowland [14] explained the multisensory activation in the SC where the inputs from cross-modal stimuli that are spatially and temporally coincident assemble onto common multisensory neurons and transform these unisensory signals into a synthesized multisensory product in which physiological responses are faster, more reliable and more robust than those elicited by either individual stimulus.

This effect is in agreement with the results on human studies of patients with HH, revealing that the spared retinotectal functions of SC neurons can be trained by AVT to enhance the multisensory integration responses and increase the visual detection of stimuli presented in the blind field through the cross-modal blindsight phenomenon [12, 18, 24, 26, 27, 31]. Interestingly, it has been shown that in the multisensory mechanism, unseen visual stimuli in HH can influence perception in other sensory modalities such as improving auditory localization [32]. In addition, this result was observed for both HH and neglect patients after the exposure to spatially coincident visual and auditory stimuli, exhibiting a significant reduction in auditory localization errors [33]. On the other hand, Frassinetti et al. [13] found direct and short-term effects of multisensory stimulation, by adding a coincident sound, on the enhancement of detection of visual targets in the affected hemifield. In a study performed on HH cats that provided specially or temporally noncongruent AVT, rehabilitation failed in all cases even when the number of training trials was twice than the number usually required for recovery [39]. In contrast, the authors demonstrated resolution of HH when the test was repeated with spatially and temporally congruent AVT. It has been assumed that rehabilitation required the neural signals from different modalities to converge onto their target neurons in the SC within a short time window in which they would be able to interact [39].

Whilst most studies detected the short-term beneficial effects of bimodal stimulation, some studies provided evidence that these effects can be persistent, up to 1 year after treatment [12, 15, 26, 27]. The exact mechanism underlying these effects is not totally clear; however, either restoration of vision or compensation is suggested. Reviewing the involved studies confirmed that by using multisensory stimulation, a direct effect of compensation on the oculomotor function enhanced the attention to the stimulated location. Grasso et al. [27] emphasized that the stability at the follow-up session on the enhancement at the clinical, oculomotor and electrophysiological levels is extremely relevant to the neural plasticity of the visual system and the multisensory integration through the SC neurons (retino-colliculo-dorsal pathway).

It is worth noting that in humans, compensatory AVT has a clear advantage in improving the oculomotor exploration and visual perception, suggesting that the improvement was not due to restoration in the visual field, but rather to a compensatory activation of oculomotor system [12, 24]. On the contrary, after several weeks of training HH cats on an AVT paradigm, visual responsiveness was restored in SC neurons and behavioural responses were elicited by visual stimuli in the blind hemifield [14]. A possible explanation for that difference between humans and animals is that procedural differences resulted in different functional outcomes, e.g. differences in the contralesional exposure locations (fixed in the animal vs. variable in the human), variations in the stimuli used and/or frequency and duration of the trial sessions as well as site and extent of the lesions. In humans, when central fixation was maintained and with the absence of visual cortices to supplement ipsilateral visual function, this may have been sufficient to inhibit visual responses of the caudal SC (reacting to peripheral space) to an extent that they were insufficient to generate detection of the peripheral visual stimulus [14]. However, this remains an open research question.

As HH can have a great impact on functional abilities of daily life, it was important to focus on ADL measures. Although only a limited number of studies measured the effect of multisensory stimulation on activities of daily living, these studies consistently report that patients in acute and chronic stages after stroke showed significant improvement on the ADL scale [12, 18, 26, 27]. Additionally, only patients who had received AVT showed near-normal daily living activities in relation to visual impairment after 3 weeks of training compared to patients trained on visual stimulation only [18]. Yet, there were no patients who received no training at all in this study, making the finding unclear about the recovery as the selected groups were in the acute stage after stroke.

In everyday life, patients with HH experience asymmetric visual inputs, leading to an imbalance of attentional bias towards the intact hemifield [32, 33]. This attentional imbalance as well as the clinical signs of HH has been found to be diminished by the bimodal training. Stimulating the multisensory integration of the SC which has a crucial role in controlling both overt and covert spatial attention might explain this effect of improvement. Schneider and Kastner [40] used high-resolution functional magnetic resonance imaging to measure responses in the human lateral geniculate nucleus (LGN) and SC during sustained spatial attention. The study results indicated that activity in the LGN and SC can be modulated by sustained spatial attention. Moreover, the attentional modulation in the SC was especially prominent, demonstrating its importance in spatially directing and sustaining attention.

EEG recordings showed that behavioural improvement elicited by multisensory stimulation coincided with reduced hyperactivation within the contralesional hemisphere. The amplitude of the P3 EEG component, which has been demonstrated to be modulated by the attention, showed a reduction after AVT, reflecting less visual spatial attention allocated to the intact hemifield [31]. In addition, by assessing the auditory and visual straight-ahead directions in patients with HH, it was found that the visual space perception, which is known to be distorted in patients with HH, was shifted towards the anopic side [35]. However, patients’ auditory perceived ‘straight ahead’ was approximately veridical, indicating that HH did not influence the supra-modal processing of body orientation in space but restricted to the visual modality only [35]. Thus, it seems as if the straight-ahead perception in relation to visual space been affected by the damaged visual areas in HH, and these areas were proposed as not contributing in a multimodal or supra-modal straight-ahead perception. Lewald et al. [34] stated that there is a high possibility to exploit the auditory system in rehabilitation of visual field deficits as patients with HH retain an undistorted representation of auditory space.

The possibility of utilizing multisensory integration to compensate for visual field defect in children and adolescents was extremely rare. Tinelli et al. [21] demonstrated amelioration of performance after AVT with improvement in the visual search behaviour, as documented by reduction of visual scanning times, which was compatible with the results published on adults. The training period required to show an improvement in children was longer than that in adults as less daily sessions were used. The authors explained that children were not able to maintain attention for a long period as adults and, in addition, motivation to achieve results was less understood by children. It has been suggested that it would be useful to apply a fMRI paradigm before and after the training, to verify what happens at the cortical level, in the damaged and intact hemispheres, after the multisensory activation in patients with visual field defect during childhood [21].

There is evidence to suggest that combining different sensory modalities can provide an effective rehabilitation method for patients with visual field deficits. Nevertheless, the neural underpinnings of the compensation of visual field loss after AVT needs further study. Systematic AVT may have improved the processing of visual information by recruiting subcortical pathways, and because most of the patients with visual cortex damage have an intact SC, it might be possible to train the use of retinotectal functions by the bimodal AVT [18]. Using functional and structural MRI techniques may help in providing more evidence for such a hypothesis. A wide range of evidence converges on the presence of two pathways for conscious and unconscious perceptions, which diverge at early processing stages. In the pathway underlying conscious visual processing, the visual information is projected from the retina to the lateral geniculate nucleus, then to the occipital lobe. Unconscious visual processing, on the other hand, relies on a second pathway, in which the visual information is projected from the retina to the SC and the pulvinar, then to the dorsal parietal cortices [27]. Tamietto et al. [41] demonstrated anatomical connections between the SC and amygdala via the pulvinar by using diffusion tensor imaging (DTI) in humans. The phenomenon of cross-modal blindsight could be explained by this alternative connection, including the SC and its dorsal parietal projections, which improve perception when multisensory cues are used.

Additionally, Hertz and Amedi [42] performed a fMRI study on healthy subjects that involved a multisensory experimental condition. It was found that there is a network of areas showing AV interaction responses including the parietal lobe, the temporal lobe, the frontal lobe and the insula [42]. In line with this concept, neuroanatomical and neurophysiological studies in animals have illustrated strong connections between SC, posterior parietal cortex and frontal eye fields for control of eye movements [43]. Furthermore, neuroimaging data proved that the preparation and execution of eye movements have been enhanced by the activation of the frontal eye field, which involves descending input pathways to the SC [44].

In conclusion, this review supports the concept that compensatory AVT can be useful as a rehabilitation method for stroke survivors with HH. Moreover, there is a considerable lack of studies using AVT stimulation on human HH patients with concurrent functional and structural MRI to identify the optimal training paradigm and the neural mechanisms underlying its effect. Further research is warranted to explore these aspects.

### Electronic supplementary material

Below is the link to the electronic supplementary material.

Supplementary file1 (PDF 149 KB)

Supplementary file2 (PDF 49 KB)

Supplementary file3 (PDF 36 KB)

Supplementary file4 (PDF 49 KB)

## Data Availability

All data generated or analysed during this study are included in this published article (and its supplementary information files).

## Appendix

Table 3

Table 4

**Table 3:** PRISMA checklist

Section/topic	#	Checklist item
**Title**		
Title	1	Identify the report as a systematic review, meta-analysis, or both
**Abstract**		
Structured summary	2	Provide a structured summary including, as applicable, background; objectives; data sources; study eligibility criteria, participants and interventions; study appraisal and synthesis methods; results; limitations; conclusions and implications of key findings; and systematic review registration number
**Introduction**		
Rationale	3	Describe the rationale for the review in the context of what is already known
Objectives	4	Provide an explicit statement of questions being addressed with reference to participants, interventions, comparisons, outcomes and study design (PICOS)
**Methods**		
Protocol and registration	5	Indicate if a review protocol exists, if and where it can be accessed (e.g. Web address), and, if available, provide registration information including registration number
Eligibility criteria	6	Specify study characteristics (e.g. PICOS, length of follow-up) and report characteristics (e.g. years considered, language, publication status) used as criteria for eligibility, giving rationale
Information sources	7	Describe all information sources (e.g. databases with dates of coverage, contact with study authors to identify additional studies) in the search and date last searched
Search	8	Present full electronic search strategy for at least 1 database, including any limits used, such that it could be repeated
Study selection	9	State the process for selecting studies (i.e. screening, eligibility, included in systematic review, and, if applicable, included in the meta-analysis)
Data collection process	10	Describe method of data extraction from reports (e.g. piloted forms, independently, in duplicate) and any processes for obtaining and confirming data from investigators
Data items	11	List and define all variables for which data were sought (e.g. PICOS, funding sources) and any assumptions and simplifications made
Risk of bias in individual studies	12	Describe methods used for assessing risk of bias of individual studies (including specification of whether this was done at the study or outcome level), and how this information is to be used in any data synthesis
Summary measures	13	State the principal summary measures (e.g. risk ratio, difference in means)
Synthesis of results	14	Describe the methods of handling data and combining results of studies, if done, including measures of consistency (e.g. I2 for each meta-analysis)
Risk of bias across studies	15	Specify any assessment of risk of bias that may affect the cumulative evidence (e.g. publication bias, selective reporting within studies)
Additional analyses	16	Describe methods of additional analyses (e.g. sensitivity or subgroup analyses, meta-regression), if done, indicating which were pre-specified
**Results**		
Study selection	17	Give numbers of studies screened, assessed for eligibility and included in the review, with reasons for exclusions at each stage, ideally with a flow diagram
Study characteristics	18	For each study, present characteristics for which data were extracted (e.g. study size, PICOS, follow-up period) and provide the citations
Risk of bias within studies	19	Present data on risk of bias of each study and, if available, any outcome level assessment (see item 12)
Results of individual studies	20	For all outcomes considered (benefits or harms), present, for each study: (a) simple summary data for each intervention group and (b) effect estimates and confidence intervals, ideally with a forest plot
Synthesis of results	21	Present the main results of the review. If meta-analyses are done, include for each, confidence intervals and measures of consistency
Risk of bias across studies	22	Present results of any assessment of risk of bias across studies (see item 15)
Additional analysis	23	Give results of additional analyses, if done (e.g. sensitivity or subgroup analyses, meta-regression [see item 16])
**Discussion**		
Summary of evidence	24	Summarize the main findings including the strength of evidence for each main outcome; consider their relevance to key groups (e.g. healthcare providers, users and policymakers)
Limitations	25	Discuss limitations at study and outcome level (e.g. risk of bias) and at review level (e.g. incomplete retrieval of identified research, reporting bias)
Conclusions	26	Provide a general interpretation of the results in the context of other evidence, and implications for future research
**Funding**		
Funding	27	Describe sources of funding for the systematic review and other supports (e.g. supply of data); role of funders for the systematic review

From Moher et al. [45]

**Table 4:** Preferred Reporting Items for Systematic Reviews and Meta-Analyses Protocols (PRISMA-P) 2015 checklist: recommended items to address in a systematic review protocol

Section and topic	Item no	Checklist item
**Administrative information**		
Title		
Identification	1a	Identify the report as a protocol of a systematic review
Update	1b	If the protocol is for an update of a previous systematic review, identify as such
Registration	2	If registered, provide the name of the registry (such as PROSPERO) and registration number
Authors		
Contact	3a	Provide name, institutional affiliation and e-mail address of all protocol authors; provide physical mailing address of corresponding author
Contributions	3b	Describe contributions of protocol authors and identify the guarantor of the review
Amendments	4	If the protocol represents an amendment of a previously completed or published protocol, identify as such and list changes; otherwise, state plan for documenting important protocol amendments
Support		
Sources	5a	Indicate sources of financial or other support for the review
Sponsor	5b	Provide name for the review funder and/or sponsor
Role of sponsor or funder	5c	Describe roles of funder(s), sponsor(s) and/or institution(s), if any, in developing the protocol
**Introduction**		
Rationale	6	Describe the rationale for the review in the context of what is already known
Objectives	7	Provide an explicit statement of the question(s) the review will address with reference to participants, interventions, comparators and outcomes (PICO)
**Methods**		
Eligibility criteria	8	Specify the study characteristics (such as PICO, study design, setting and time frame) and report characteristics (such as years considered, language and publication status) to be used as criteria for eligibility for the review
Information sources	9	Describe all intended information sources (such as electronic databases, contact with study authors, trial registers or other grey literature sources) with planned dates of coverage
Search strategy	10	Present draft of search strategy to be used for at least 1 electronic database, including planned limits, such that it could be repeated
Study records		
Data management	11a	Describe the mechanism(s) that will be used to manage records and data throughout the review
Selection process	11b	State the process that will be used for selecting studies (such as 2 independent reviewers) through each phase of the review (that is, screening, eligibility and inclusion in meta-analysis)
Data collection process	11c	Describe planned method of extracting data from reports (such as piloting forms, done independently, in duplicate), any processes for obtaining and confirming data from investigators
Data items	12	List and define all variables for which data will be sought (such as PICO items and funding sources), any pre-planned data assumptions and simplifications
Outcomes and prioritization	13	List and define all outcomes for which data will be sought, including prioritization of main and additional outcomes, with rationale
Risk of bias in individual studies	14	Describe anticipated methods for assessing risk of bias of individual studies, including whether this will be done at the outcome or study level, or both; state how this information will be used in data synthesis
Data synthesis	15a	Describe criteria under which study data will be quantitatively synthesised
	15b	If data are appropriate for quantitative synthesis, describe planned summary measures, methods of handling data and methods of combining data from studies, including any planned exploration of consistency (such as I2, Kendall’s *τ*)
	15c	Describe any proposed additional analyses (such as sensitivity or subgroup analyses and meta-regression)
	15d	If quantitative synthesis is not appropriate, describe the type of summary planned
Meta-bias(es)	16	Specify any planned assessment of meta-bias(es) (such as publication bias across studies and selective reporting within studies)
Confidence in cumulative evidence	17	Describe how the strength of the body of evidence will be assessed (such as GRADE)

The copyright for PRISMA-P (including checklist) is held by the PRISMA-P Group and is distributed under a Creative Commons Attribution Licence 4.0

## Declarations

### Systematic review registration

This review is registered with PROSPERO by the correspondence author (CRD42020197348). This article is present on a preprint server of Research Square website and can be accessed on https://www.researchsquare.com/article/rs-478721/v1. This article is not published nor is under publication elsewhere.

## Abbreviations

ADL: Activities of daily living
AV: Audio-visual
AVT: Audio-visual training
DTI: Diffusion tensor imaging
EEG: Electroencephalographic
EOG: Electro-oculography
fMRI: Functional magnetic resonance imaging
HH: Homonymous hemianopia
SC: Superior colliculus
SRB: Saccade-related burst
VM: Visual motor
VT: Visual training
