# Supplementary material for: Audio-visual stimulation for visual compensatory functions in stroke survivors with visual field defect: a systematic review
Source: Neurol Sci. 2022 Feb 11;43(4):2299–321. doi: 10.1007/s10072-022-05926-y (PMC8918177; doi:10.1007/s10072-022-05926-y)
Supplement: Supplementary file 2 — Supplementary file2 (PDF 49 KB) [file 10072_2022_5926_MOESM2_ESM.pdf]

Audio-visual stimulation for visual compensatory functions in stroke survivors with visual field defect; A systematic review

Neurological Sciences

Corresponding author:

Kholoud Alwashmi

Department of Psychological Sciences,

Eleanor Rathbone Building,

University of Liverpool, UK

Liverpool L69 3BX

E: K.Alwashmi@liverpool.ac.uk

Co-authors:

Georg Meyer

Department of Psychological Sciences,

Eleanor Rathbone Building,

University of Liverpool, UK

Liverpool L69 3BX

E: Georg@liverpool.ac.uk

Fiona J Rowe

Institute of Population Health

University of Liverpool, UK

Liverpool L69 3BX

E: Rowef@liverpool.ac.uk

SUPPLEMENTARY TABLE 1: QUALITY ASSESSMENT OF OBSERVATIONAL STUDIES USING THE STROBE CHECKLIST

		Title Abstract	Background	Objectives	Study design	Setting	Participants	Variables	Measurement	Bias	Study size	Quantitative variables	Statistical methods	Participants	Descriptive data	Outcome data	Main results	Other analyses	Key results	Limitations	Interpretation	Generalizabilit	Funding	Score
1.	**Tinelli 2017**	+	+	+	+	+	+	+	+	-	-	+	+	-	+	+	+	n/a	+	-	+	-	-	73
2.	**Passamonti 2009**	+	+	+	+	+	+	+	+	-	-	+	+	+	+	+	+	n/a	+	-	+	+	+	87
3.	**Lewald 2013**	+	+	+	+	+	+	+	+	-	-	+	+	-	+	+	+	n/a	+	+	+	-	-	78
4.	**Frassinetti 2005**	+	+	+	+	+	+	+	+	-	-	+	+	-	+	+	+	n/a	+	-	+	+	+	82
5.	**Lewald 2012**	+	+	+	+	+	+	+	+	+	-	+	+	-	+	+	+	n/a	+	+	+	-	+	87
6.	**Dundon 2015**	+	+	+	+	+	+	+	+	-	-	+	+	-	+	+	+	n/a	+	-	+	-	+	77
7.	**Passamonti 2009**	+	+	+	+	+	+	+	+	-	-	+	+	-	+	+	+	n/a	+	-	+	-	+	76
8.	**Tinelli 2015**	+	+	+	+	+	+	+	+	-	-	+	+	+	+	+	+	n/a	+	+	+	-	+	87
9.	**Grasso 2016**	+	+	+	+	+	+	+	+	-	-	+	+	-	+	+	+	n/a	+	-	+	+	+	82
10.	**Leo 2008**	+	+	+	+	+	+	+	+	-	-	+	+	-	+	+	+	n/a	+	-	+	-	+	76
11.	**Lewald 2009**	+	+	+	+	+	+	+	+	-	-	+	+	-	+	+	+	n/a	+	-	+	-	-	73
12.	**Ten Brink 2015**	+	+	+	+	+	+	+	+	-	-	+	+	-	+	+	+	n/a	+	+	+	-	-	77
