# Supplementary material for: Audio-visual stimulation for visual compensatory functions in stroke survivors with visual field defect: a systematic review
Source: Neurol Sci. 2022 Feb 11;43(4):2299–321. doi: 10.1007/s10072-022-05926-y (PMC8918177; doi:10.1007/s10072-022-05926-y)
Supplement: Supplementary file 3 — Supplementary file3 (PDF 36 KB) [file 10072_2022_5926_MOESM3_ESM.pdf]

Audio-visual stimulation for visual compensatory functions in stroke survivors with visual field defect; A systematic review

Neurological Sciences

Corresponding author:

Kholoud Alwashmi

Department of Psychological Sciences,

Eleanor Rathbone Building,

University of Liverpool, UK

Liverpool L69 3BX

E: K.Alwashmi@liverpool.ac.uk

Co-authors:

Georg Meyer

Department of Psychological Sciences,

Eleanor Rathbone Building,

University of Liverpool, UK

Liverpool L69 3BX

E: Georg@liverpool.ac.uk

Fiona J Rowe

Institute of Population Health

University of Liverpool, UK

Liverpool L69 3BX

E: Rowef@liverpool.ac.uk

SUPPLEMENTARY TABLE 2: QUALITY ASSESSMENT OF RANDOMISED TRIALS USING THE CONSORT CHECKLIST

Section/Topic	Checklist item	Item No	Keller 2010	Bolognini 2005
Title and abstract	Identification as a randomised trial in the title	1a	-	-
	Structured summary of trial design, methods, results, and conclusions	1b	+	+
Introduction	Objectives	2b	+	+
Methods	Trial design	3a	+	+
	Changes to methods	3b	-	-
	Eligibility	4a	+	+
	Interventions for each group	5	+	+
	Outcome measures	6a	+	+
	Changes to trial outcomes	6b	-	-
	Sample size	7a	-	-
	Interim analysis	7b	N/A	N/A
	Method of random allocation sequence	8a	+	-
	Randomisation	8b	+	-
	Implementation of random allocation	9	+	-
	Generation of random allocation	10	-	-

	Blinding	11a	+	-
	Similarity of interventions	11b	+	+
	Statistical methods	12a	+	+
	Additional analyses	12b	+	+
Results	No. of participants	13a	+	+
	Losses and exclusions	13b	+	+
	Dates of recruitment - follow up	14a	+	+
	Reason trial ended	14b	N/A	N/A
	Baseline demographic	15	+	+
	Analysis of original assigned groups	16	+	+
	Results with precision	17a	+	+
	Additional analysis	18	+	+
	Harms	19	-	-
Discussion	Limitations	20	+	-
	Generalisability	21	+	+
	Consistent interpretation	22	+	+
Other Info	Registration	23	-	-

	Access to full protocol	24	-	-
	Funding	25	+	+
Overall %			81	62
