# Supplementary material for: Audio-visual stimulation for visual compensatory functions in stroke survivors with visual field defect: a systematic review
Source: Neurol Sci. 2022 Feb 11;43(4):2299–321. doi: 10.1007/s10072-022-05926-y (PMC8918177; doi:10.1007/s10072-022-05926-y)
Supplement: Supplementary file 4 — Supplementary file4 (PDF 49 KB) [file 10072_2022_5926_MOESM4_ESM.pdf]

Audio-visual stimulation for visual compensatory functions in stroke survivors with visual field defect; A systematic review

Neurological Sciences

Corresponding author:

Kholoud Alwashmi

Department of Psychological Sciences,

Eleanor Rathbone Building,

University of Liverpool, UK

Liverpool L69 3BX

E: K.Alwashmi@liverpool.ac.uk

Co-authors:

Georg Meyer

Department of Psychological Sciences,

Eleanor Rathbone Building,

University of Liverpool, UK

Liverpool L69 3BX

E: Georg@liverpool.ac.uk

Fiona J Rowe

Institute of Population Health

University of Liverpool, UK

Liverpool L69 3BX

E: Rowef@liverpool.ac.uk

SUPPLEMENTARY TABLE 3: QUALITY ASSESSMENT OF REVIEWS USING THE PRISMA CHECKLIST

Section/Topic	Checklist item	Item No	Tinga 2016	Dundon 2015
Title	Identify the report as a systematic review, meta-analysis, or both.	1	+	-
Abstract	Provide a structured summary including, as applicable: background; objectives; data sources; study eligibility criteria, participants, and interventions; study appraisal and synthesis methods; results; limitations; conclusions and implications of key findings; systematic review registration number.	2	+	+
Introduction	Rationale	3	+	+
	Objectivs	4	+	+
Methods	Protocol and registration	5	+	-
	Eligibility criteria	6	+	-
	Information sources	7	+	-
	Search	8	+	-
	Study selection	9	+	-
	Data collection process	10	+	-
	Data items	11	+	-
	Risk of bias in individual studies	12	+	+
	Summary measures	13	-	-

	Synthesis of results	14	-	-
	Risk of bias across studies	15	+	-
	Additional analyses	16	-	-
Results	Study selection	17	+	-
	Study characteristics	18	+	-
	Risk of bias within studies	19	+	+
	Results of individual studies	20	+	+
	Synthesis of results	21	+	+
	Risk of bias across studies	22	+	+
	Additional analysis	23	-	-
Discussion	Summary of evidence	24	+	+
	Limitations	25	+	-
	Conclusions	26	+	+
Other	Funding	27	+	+
Overall %			86	52
